# The Scope of Inquiry and the Illusions of Low Expectations: A Commentary on “Illusions of Understanding in the Sciences”

**DOI:** 10.1007/s42113-026-00280-0

**Published:** 2026-04-20

**Authors:** Philipp Koralus, Vincent Wang-Mascianica, Marco Ragni

**Affiliations:** 1 HAI Lab, University of Oxford, Oxford, UK; 2 Predictive Analytics, Chemnitz University of Technology, Chemnitz, Germany

Shiffrin, Stigler, and Keil argue that the sciences are prone to illusions of understanding. We agree. However, we suggest that many such illusions do not arise from overconfidence alone, but from lack of clarity about the broader background question that provides the scaffolding for particular studies. A model may successfully describe observed data, predict new observations, guide successful causal intervention, or even provide the foundation for a new technology. All of these milestones are distinctive achievements. Aiming for them involves guiding particular studies with the appropriate background question so that they ultimately add up to an answer that delivers the milestone.

What background question is implicitly chosen may limit our inquiry to only some of these milestones. It is not only the case that any particular study conducted by an individual scientist at a time is limited in scope; it is also the case that sometimes the entire horizon of inquiry becomes limited and the bar for what counts as understanding gets lowered. Indeed, for many areas of science, including psychology, the more challenging milestones of scientific inquiry are for now systematically out of reach. This limitation makes it easy to fall for an illusion of understanding by settling into low expectations. If even an entire branch department of a science cannot move beyond limited milestones after working for decades, we might come to ignore that other milestones exist partly because we would otherwise have to conclude that our contributions to understanding have been small. Our broader background question about what milestones a branch of science can aim for in practice shapes what further questions our scientific investigations are taken to be “on the hook” for. Mistaking equilibrium of judgment with respect to the questions appropriate for one type of milestone of scientific inquiry with equilibrium with respect to the questions involved in pursuing a more demanding one produces the appearance of greater understanding than is justified.

Scientific inquiry is an application of reason and the erotetic theory provides a useful framework for understanding it. According to it, normatively correct reasoning yields judgments that are robust across an appropriate range of further questions (Koralus 2023). Part of the decentralized genius of the scientific tradition is that it has managed to continuously expand the range of questions that we can collectively recognize as demanding consideration if we are going to form certain judgments, without falling into the skeptical trap of treating all putatively relevant questions as potentially blocking judgment. For example, our judgment about a putative empirical result in psychology must be robust to the question of whether experimenter effects could explain the data. At the same time, we understand that we are not on the hook for empirical judgments to be robust to the question of whether we could be deceived by a Cartesian demon or whether we have evidence to conclude that the future will resemble the past. These questions are excluded not because the “handmaidens to science” in the philosophy department have answered them, but because rational empirical inquiry into particular empirical questions does not have to be in equilibrium with respect to skeptical scenarios. Further questions about skeptical scenarios do not normally change what answers we give to any particular empirical inquiries, since they threaten them all equally.

Through this lens, a published paper is not merely a report of results. It is implicitly a claim that a certain form of erotetic equilibrium has been reached. What counts as such equilibrium depends on the ambition of the inquiry: whether one seeks description, prediction, mechanism, intervention, or reconstruction. Just as empirical science sets aside radical skepticism, research communities may set aside more demanding empirical questions - “can we control the causal process?”, “do we understand what grounds this phenomenon?”, “can we rebuild this phenomenon as a technology?” - as outside the scope of scientific understanding. The crucial difference is that, in empirical domains, increased causal control and the ability to translate theory into technology typically correspond to deeper understanding. Where such ambitions are ignored, equilibrium may be achieved only relative to a weakened question-set. Unlike the case of skeptical scenarios, asking, for example, whether a certain proposed model of a phenomenon would enable us to recreate it as a form of technology *does* potentially change what answer we might give to a particular empirical inquiry.

Differences in the scope of questions can be seen even between sub-areas of one discipline like psychology: In experimental psychology, researchers manipulate independent variables and use statistics to establish relations between hypotheses and results. In many cases, establishing a statistically reliable association is considered a sufficient answer. By contrast, cognitive psychology aims to specify formalized cognitive processes, often algorithmically, that generate behavior over time. The demand here is not merely descriptive adequacy but process-level explanation. Model components must correspond to experimentally accessible structures, aligning interventions in the model with interventions in the cognitive system. The relevant question shifts from “What relation holds?” or even “How does it arise?” to “What would happen if we selectively altered component X?” This introduces counterfactual robustness as a constitutive demand. Unified cognitive architectures (e.g., ACT-R) realize this by integrating multiple cognitive functions within a single framework capable of accounting for reaction times, error patterns, learning curves, etc. These differences reveal a crucial point: questions are posed by researchers, but what is accepted as an answer, and what further questions this answer needs to be robust to, is regulated by disciplinary norms, methodological standards, and reviewer expectations.

From an erotetic perspective, a paper is a formal bridge between a set of questions and a judgment. Its function is to establish that a certain judgment can be held as relevantly in equilibrium relative to an appropriate question-set or “inquiry complex” (Koralus 2025). Such a judgment is then generally considered in the field as contributing to or constituting a form of “understanding”. Whether a logistic regression is “enough”, whether out-of-sample prediction is required, or whether a cognitively interpretable mechanism must be specified depends on what inquiry complex our field takes to determine our standards for equilibrium of judgment. These conditions can be ordered by strength. Illusions of understanding arise when stabilization at one level is rhetorically or implicitly treated as stabilization at a stronger one.

## Data Availability

No datasets were generated or analysed during the current study.
